# Cord blood transfusions in extremely low gestational age neonates to reduce severe retinopathy of prematurity: results of a prespecified interim analysis of the randomized BORN trial

**DOI:** 10.1186/s13052-024-01714-w

**Published:** 2024-08-07

**Authors:** Luciana Teofili, Patrizia Papacci, Carlo Dani, Francesco Cresi, Giulia Remaschi, Claudio Pellegrino, Maria Bianchi, Giulia Ansaldi, Maria Francesca Campagnoli, Barbara Vania, Domenico Lepore, Fabrizio Gaetano Saverio Franco, Marco Fabbri, Roberta Penta de Vera d’ Aragona, Anna Molisso, Enrico Beccastrini, Antonella Dragonetti, Lorenzo Orazi, Tina Pasciuto, Iolanda Mozzetta, Antonio Baldascino, Emanuela Locatelli, Caterina Giovanna Valentini, Carmen Giannantonio, Brigida Carducci, Sabrina Gabbriellini, Roberto Albiani, Elena Ciabatti, Nicola Nicolotti, Silvia Baroni, Alessandro Mazzoni, Federico Genzano Besso, Francesca Serrao, Velia Purcaro, Alessandra Coscia, Roberta Pizzolo, Genny Raffaeli, Stefania Villa, Isabella Mondello, Alfonso Trimarchi, Flavia Beccia, Stefano Ghirardello, Giovanni Vento

**Affiliations:** 1 Fondazione Policlinico A. Gemelli IRCCS, Largo Gemelli 8, 00168 Roma, Italy; 2 Università Cattolica del Sacro Cuore, Roma, Italy; 3 Azienda Ospedaliero Universitaria Careggi, Firenze, Italy; 4 Città della Salute e della Scienza, Torino, Italy; 5 Department of Public Health and Pediatrics, Università di Torino, Torino, Italy; 6 Azienda Ospedaliero Universitaria Pisana, Pisa, Italy; 7 Ospedale Santobono Pausilipon, Napoli, Italy; 8 Ospedale Evangelico Villa Betania, Napoli, Italy; 9 Polo Nazionale Ipovisione IAPB Italia Onlus, Roma, Italy; 10 Fondazione IRCCS Ca’ Granda Ospedale Maggiore Policlinico, Milano, Italy; 11 Department of Clinical Sciences and Community Health, Università di Milano, Milano, Italy; 12 Azienda Ospedaliera Bianchi Melacrino Morelli, Reggio Calabria, Italy; 13 Fondazione IRCCS Policlinico S. Matteo, Pavia, Italy

**Keywords:** ELGAN, ROP, Transfusions, HbF, Umbilical blood, Randomized controlled trial

## Abstract

**Background:**

Preterm infants are at high risk for retinopathy of prematurity (ROP), with potential life-long visual impairment. Low fetal hemoglobin (HbF) levels predict ROP. It is unknown if preventing the HbF decrease also reduces ROP.

**Methods:**

BORN is an ongoing multicenter double-blinded randomized controlled trial investigating whether transfusing HbF-enriched cord blood-red blood cells (CB-RBCs) instead of adult donor-RBC units (A-RBCs) reduces the incidence of severe ROP (NCT05100212). Neonates born between 24 and 27 + 6 weeks of gestation are enrolled and randomized 1:1 to receive adult donor-RBCs (A-RBCs, arm A) or allogeneic CB-RBCs (arm B) from birth to the postmenstrual age (PMA) of 31 + 6 weeks. Primary outcome is the rate of severe ROP at 40 weeks of PMA or discharge, with a sample size of 146 patients. A prespecified interim analysis was scheduled after the first 58 patients were enrolled, with the main purpose to evaluate the safety of CB-RBC transfusions.

**Results:**

Results in the intention-to-treat and per-protocol analysis are reported. Twenty-eight patients were in arm A and 30 in arm B. Overall, 104 A-RBC units and 49 CB-RBC units were transfused, with a high rate of protocol deviations. A total of 336 adverse events were recorded, with similar incidence and severity in the two arms. By per-protocol analysis, patients receiving A-RBCs or both RBC types experienced more adverse events than non-transfused patients or those transfused exclusively with CB-RBCs, and suffered from more severe forms of bradycardia, pulmonary hypertension, and hemodynamically significant patent ductus arteriosus. Serum potassium, lactate, and pH were similar after CB-RBCs or A-RBCs. Fourteen patients died and 44 were evaluated for ROP. Ten of them developed severe ROP, with no differences between arms. At per-protocol analysis each A-RBC transfusion carried a relative risk for severe ROP of 1.66 (95% CI 1.06–2.20) in comparison with CB-RBCs. The area under the curve of HbF suggested that HbF decrement before 30 weeks PMA is critical for severe ROP development. Subsequent CB-RBC transfusions do not lessen the ROP risk.

**Conclusions:**

The interim analysis shows that CB-RBC transfusion strategy in preterm neonates is safe and, if early adopted, might protect them from severe ROP.

**Trial registration:**

Prospectively registered at ClinicalTrials.gov on October 29, 2021. Identifier number NCT05100212.

**Supplementary Information:**

The online version contains supplementary material available at 10.1186/s13052-024-01714-w.

## Background

Progressive improvements in obstetric and neonatal care have yielded significant decreases in mortality of preterm infants, particularly for those born at 23–25 weeks of gestational age [1]. Nonetheless, there are concerns regarding long-term morbidities faced by the surviving infants [2]. Among various complications, the incidence of retinopathy of prematurity (ROP) has recently remained stable or even increased [3, 4]. ROP is a leading cause of childhood blindness and significantly influences neurodevelopmental outcomes of affected patients [5, 6]. The pathogenesis of ROP involves abnormal angiogenesis in the immature retina, with low gestational age and birth weight as primary risk factors [7]. An association between fetal hemoglobin (HbF) reduction and ROP incidence was also reported, likely explaining the connection between red blood cell (RBC) transfusions and ROP [8–10].

RBC transfusions are acknowledged causes of oxidative stress in preterm neonates. Following RBC transfusions, non-transferrin bound iron has been reported to increase in preterm but not in term neonates [11]. Similarly, after transfusions, a raise of markers of hemolysis, inflammation and endothelial cell activation occurs [12–14]. In comparison with non-transfused similar-age neonates, preterm neonates receiving repeated RBC transfusions experience prolonged exposure to adult hemoglobin (HbA) [15, 16]. The lower oxygen affinity of HbA compared to HbF likely perturbs physiological oxygen delivery to developing organs and tissues and was proposed to play a role in ROP development and progression [17]. Accordingly, preterm neonates with lower HbF show significantly higher oxidative stress biomarkers levels [18]. Allogenic cord blood (CB) collected from full-term deliveries is a suitable source of HbF-enriched RBCs (CB-RBCs) for transfusion into preterm neonates. In a pilot proof-of-concept study enrolling extremely low birth weight neonates, CB-RBC concentrates increased Hb levels as well as RBC concentrates from adult donors (A-RBCs), without decreasing HbF levels [19].

BORN is a randomized, multi-center, double-blinded, controlled trial to assess whether transfusing CB-RBCs, instead of A-RBCs, reduces the incidence of severe ROP in extremely low gestational age neonates (ELGANs, i.e., neonates born at less than 28 weeks of gestation) [19]. The current study reports the results of the prespecified interim analysis on the safety of this transfusion strategy and, secondarily, on the efficacy in preventing severe ROP.

## Methods

### Study aim, design and participants

BORN is an ongoing randomized, multi-center, double-blinded, controlled trial investigating CB-RBC instead of A-RBC transfusion to reduce the incidence of severe ROP. A detailed version of the study protocol has been previously published [20]. Inclusion criteria are birth at a gestational age (GA) between 24 + 0 and 27 + 6 weeks and signed informed consent of parents or legal representatives. Out-born infants are suitable for enrolment only if not previously transfused. Exclusion criteria include maternal-fetal immunization, hydrops fetalis, major congenital malformations associated, or not, with genetic syndromes [21], previous transfusions, hemorrhage at birth, congenital infections, and the health care team deeming it inappropriate to approach the infant’s family for informed consent. The study is approved by the Ethics Committee of Fondazione Policlinico A. Gemelli IRCCS (ID 4364, Prot. N. 003590/21) and of all participating centers, and is registered at https://clinicaltrials.gov (NCT05100212).

### Enrollment, randomization and masking

Neonates are enrolled at NICU admission and randomized 1:1 to receive either standard A-RBCs (Arm A, control) or CB-RBCs (Arm B, intervention) until the post-menstrual age (PMA) of 31 + 6 weeks, with subsequent transfusions consisting of only standard A-RBCs. Twins are assigned to the same arm [22]. If compatible CB-RBC units are not available, A-RBC units are assigned. RBC types are given according to the arm allocation until the PMA of 31 + 6 weeks. The allocation sequence is generated using both stratifications for center and gestational age (< or ≥ 26 weeks), and permuted blocks with random block sizes and block order, using the NCSS 2020 Statistical Software 2020 (NCSS, LLC. Kaysville, UT, USA, ncss.com/software/ncss). The allocation table is not disclosed to ensure concealment, and randomization is provided through the Research Electronic Data Capture (RedCap) (RRID: SCR_003445) electronic data capture tool, hosted at Fondazione Policlinico Universitario A. Gemelli, IRCCS (https://redcap-irccs.policlinicogemelli.it/). Randomization is performed by the blood bank medical staff; treating neonatologists are unaware to which arm neonates were assigned. To conceal the type of blood product, A-RBCs and CB-RBCs are distributed to neonatal intensive care units (NICUs) in the same type of bags (CompoFlex 4 F RCC storage pediatric bags).

### Intervention and control description

Enrolled patients receive standard A-RBCs (Arm A, control) or allogenic CB-RBCs (Arm B, intervention). CB units are provided from public cord blood banks belonging to the Italian Cord Blood Bank Network, and processed into CB-RBC concentrates according to a standardized procedure detailed in the protocol study [20]. Both CB-RBC and A-RBC concentrates are pre-storage leukodepleted and prepared in compliance with quality standards required by Italian regulations and European guidelines. CB-RBC units are proved negative for bacterial and fungal contamination before distribution. All units are matched for ABO/RhD antigens, and γ-irradiated at the time of distribution. Transfusion therapy is managed according to Italian transfusion guidelines, and erythropoietin is administered as per center procedure [22].

### Study outcome

The primary outcome of BORN is the incidence of severe ROP (stages 3 or higher according to International Classification criteria) [23] in the CB-RBC and A-RBC arms at discharge or at 40 weeks of PMA, whichever occurs first. ROP assessment is performed through indirect ophthalmoscopy, and RetCam imaging is used to confirm the diagnosis and to monitor disease status and treatment. According to the primary outcome, a sample size of 146 patients (73 per arm) was estimated. Due to the innovative type of the intervention, an interim analysis was planned after the first 58 patients were randomized, to confirm the safety of CB-RBC transfusion in ELGANs.

### Data collection

Data are collected in REDCap in different record forms for patients and for CB unit processing. Baseline patient data include: demographics, obstetric pathology, gestational age, birth weight, antenatal prophylaxis for hyaline membrane disease, suspected (maternal fever, leukocytosis, uterine tenderness, malodorous discharge, tachycardia) or documented (histology) chorioamnionitis, post-natal steroid administration, hematological parameters [hemoglobin concentration (Hb), hematocrit (Htc), and HbF (assessed spectrophotometrically using point-of-care blood gas analyzers and expressed as percentage of total Hb), Apgar index at 1 and 5 min, Clinical Risk Index for Babies-II (CRIB-II) score, and mortality probability [24]. During hospitalization, the following clinical variables are recorded: hemodynamically-significant patent ductus arteriosus (hsPDA), maximal stage of ROP [23], necrotizing enterocolitis (NEC) [25, 26], bronchopulmonary dysplasia (BPD) [27], intraventricular hemorrhage (IVH) [28], ROP treatment, erythropoietin administration, microbiologically-documented infections, duration of oxygen therapy, ventilation support (i.e., invasive ventilation: high frequency oscillatory ventilation and/or other conventional ventilation modalities; non-invasive ventilation: continuous positive airway pressure and/or high flow nasal cannula), surgery, and death. All complications during the clinical course were considered adverse events: the severity was reported according to the Common Terminology Criteria for Adverse Events (CTCAE) v4.0, ranging from 1 (no symptoms) to 5 (death), while the imputability to previous transfusions was scored according to the European regulations on transfusion surveillance from 0 to 3, where 0 indicates excluded/unlikely, 1 possible, 2 likely/probable, and 3 certain. Hb, Hct, and HbF values were recorded twice per week. At each RBC transfusion, the following were recorded: unit identifier number, date of transfusion, Hct value before and after transfusion, and post-transfusion (within 24 h) pH, lactate, and potassium levels. Data collected for each CB-RBC unit included parameters at: collection (collection date and CB volume before fractionation), processing (date, post-processing RBC recovery, platelet depletion, residual leukocyte count, hemolysis rate), and, for non-transfused units, the end-of-storage hemolysis rate (at 14 days).

### Statistical analysis

A comprehensive descriptive analysis of the study population was performed. Demographic characteristics, clinical features, and relevant baseline variables were expressed as median and interquartile range (IQR), mean and standard deviation (SD), or proportions. To investigate differences between groups, we employed the Chi2 test, applying Fisher correction if needed. Relative Risk (RR) was calculated to assess the association between the RBC-type transfusion and the primary outcome (ROP). The impact of transfusion burden on the primary outcome was estimated by logistic regression analysis, using the primary outcome as a dependent variable. This method allowed us to assess the influence of the RBC type, adjusting for the number of transfusions and other covariates selected based on their clinical relevance and significance in univariate analyses. Adjusted odds ratios (ORs) and their corresponding confidence intervals were reported to quantify the association between the predictors and the outcome. To explore the impact of blood products on HbF levels, the area under the curve (AUC) of HbF and PMA was calculated, and results were compared among different groups of patients. Missing data, unless not otherwise specified, were below 5% and were not managed except for the AUC calculation of HbF and PMA. In this case, whenever possible, missing data were calculated based on the preceding HbF determination using previously reported algorithms [19].

Statistical analysis was performed in the “intention-to-treat” set (all enrolled patients, independently if they were or not transfused, including major protocol deviations, i.e., those patients in the CB-RBC arm who received A-RBC transfusions due to CB-RBC unit unavailability), and “per protocol” set (all patients categorized according to the transfusions received until the PMA of 32 weeks: no transfusion, only A-RBCs, only CB-RBCs, both A-RBCs and CB-RBCs).

The following software programs were used to perform statistical analysis and prepare illustrations: NCSS 10 Statistical Software 2015 (NCSS, LLC. Kaysville, Utah, USA, ncss.com/software/ncss), Stata Statistical Software, Release 18 (StataCorp. 2023. College Station, TX: StataCorp LLC) and GraphPad Prism version 10.0.0 (GraphPad Software, Boston, Massachusetts USA, www.graphpad.com).

## Results

**Fig. 1:**
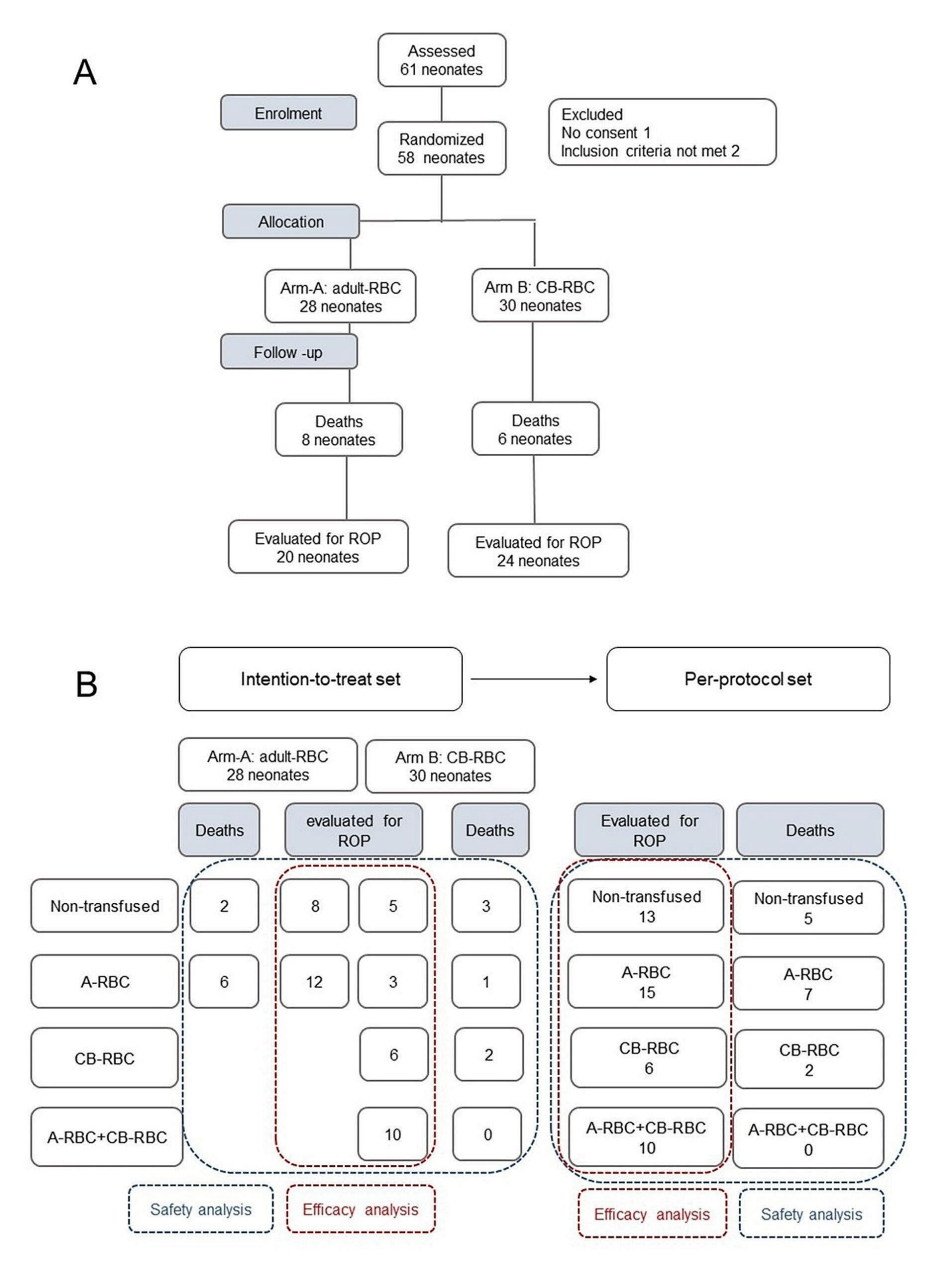
Study population entering the interim analysis of safety of CB-RBC transfusions and of impact on the rate of severe ROP. (**A**) Flow-diagram of the study. (**B**) For the high rate of protocol deviations, a parallel analysis of safety and efficacy of CB-RBC transfusions was performed in the ”intention-to-treat” and “per protocol” settings

Study recruitment started on December 1, 2021, and the first patient was enrolled on December 17, 2021. Patients were recruited in NICUs of 4 tertiary Italian hospitals, and CB-RBC units were collected by 6 public CB banks. By August 31, 2023, 58 patients were enrolled and completed their follow-up: 14 died without reaching the primary endpoint (40 weeks of PMA or discharge, whichever occurred first), whereas 44 reached the primary endpoint and were evaluated for ROP (Fig. 1A). Figure 1B shows the patients’ distribution according to the types of blood products received and the two sets of analysis. Table 1 illustrates clinical and laboratory characteristics of the 58 patients: 28 were assigned to the arm A (A-RBCs) and 30 to the arm B (CB-RBCs). The arms were well balanced for prenatal and neonatal characteristics, hematological parameters at birth, and follow-up duration. Four patients in the Arm A and 6 in the arm B received erythropoietin during hospitalization, that was administered intravenously at a dose of 200 U/kg/die, followed by a maintenance dose of 400 U/Kg by subcutaneous injection three times per week. The overall mortality was 24.1% (95% CI 15.0-36.6) and the mortality rate was similar in the two arms (Table 1). Moreover, the mortality rate did not statistically differ among groups regardless of the type of transfusions received [31.3% (95% CI 14.2–55.6) in non-transfused patients, 29.2% (95% CI 14.9–49.2) in neonates receiving only A-RBCs, 25.0% (95% CI 4.4–59.1) in those receiving only CB-RBCs, and 0 (95% CI 0-27.8) in those receiving both A-RBCs and CB-RBCs, *p* = 0.235)].

**Table 1:** Clinical characteristics at birth and relevant antenatal features of the study population

	All patients *n* = 58	Arm A *n* = 28	Arm B *n* = 30	*P*
**Gestational age**,** weeks**	26.1 (25.3–27.1)	26.2 (25.4–27.1)	26.1 (24.9–27.4)	0.906
**Weight**,** gr**	750 (650–911)	745 (648–903)	762 (660–920)	0.454
**Time to randomization**,** days**	1 (0–3)	1 (0–4)	2 (1–4)	0.328
**Male / Female**,** n (%)**	32(55.2) /26 (44.8)	14 (50.0) /14 (50.0)	18 (60.0) /12 (40.0)	0.444
**Twins**,** n (%)**	14 (24.1)	8 (28.6)	6 (20.0)	0.445
**Apgar score 1 min**	6 (4–7)	6 (5–7)	6 (4–7)	0.378
**Apgar score 5 min**	8 (7–9)	8 (8–9)	8 (7–9)	0.432
**CRIB II score**	11 (9–13)	11 (9–13)	11 (9–13)	0.777
**Probability of mortality (%)**	17.8 (8.1–34.8)	17.8 (9.1–34.8)	17.8 (8.1–34.8)	0.771
**Hb at birth**,** g/dL**	14.9 (13.8–16.5)	15.0 (14.0-17.4)	14.6 (13.8–16.2)	0.302
**Hct at birth (%)**	44.0 (41.0-49.5)	46.0 (42.0–52.0)	44.0 (40.0-47.5)	0.277
**Obstetric pathology**				
*Preeclampsia*	4 (6.9)	3 (10.7)	1 (3.3)	0.267
*Maternal diabetes*	2 (3.4)	1 (3.6)	1 (3.3)	0.960
*PROM*	26 (44.8)	9 (32.1)	17 (56.7)	0.060
*Placenta previa*	4 (6.9)	1 (3.6)	3 (10.0)	0.334
*Placental abruption*	11(18.9)	4 (14.3)	7 (23.3)	0.379
*ARED*	6 (10.3)	1 (3.6)	5 (16.7)	0.092
*AED*	13 (22.4)	9 (32.1)	4 (13.3)	
*Suspected chorioamnionitis*	7 (12.1)	3 (10.7)	4 (13.3)	0.953
*Documented chorioamnionitis*	4 (6.9)	2 (7.1)	2 (6.7)	
**Antenatal therapy**				
*Steroid therapy*	51 (87.9)	27 (96.4)	24 (80.0)	0.054
*Complete steroid therapy*	36 (62.1)	16 (57.1)	20 (66.7)	0.455
*Antibiotics*	33 (59.6)	17 (60.7)	16 (53.3)	0.570
Complete antibiotic therapy*	21 (64.7)	8 (47.0)	13 (81.2)	0.070
*Magnesium sulphate*	38 (65.5)	20 (71.4)	18 (60.0)	0.360
**Death**,** n (%)**	14 (24.1)	8 (28.5)	6 (20)	0.545
**Follow up**,** days**	103 (63–131)	103 (24–132)	104 (63–131)	0.796

Data are given as N (%) or median (IQR). * Among 33 neonates receiving antibiotics. CRIB II score: Clinical Risk Index for Babies II score; PROM: premature rupture of membranes; ARED: absent or reversed end-diastolic flow in the umbilical artery; AED: absent end-diastolic flow in the umbilical artery

### Adverse events and imputability of previous transfusions

During the study period, 336 adverse events were recorded: 165 occurred in the arm A and 171 in the arm B (Table 2). Individual patients could have multiple different types of events or multiple episodes of the same event. There was no difference between arms regarding the incidence of any of the events listed (Table 2). The rate of adverse events was also assessed in the “per protocol” setting (eTable 1), comparing patients enrolled according to the type of blood products received before the PMA of 32 weeks (Fig. 1B). Comprehensively considered, patients receiving only A-RBCs or both types of RBCs experienced more adverse events than non-transfused neonates or those transfused with only CB-RBCs (p 0.014). A slightly higher incidence of surgery was observed in patients receiving both RBC types (*p* = 0.052), suggesting a particular complexity in the clinical course of these neonates. The severity of various adverse events was comparable between arms (Table 2). Nevertheless, when evaluated in the “per-protocol” setting, neonates receiving only A-RBCs or both types of RBCs tended to exhibit more severe forms of bradycardia (*p* = 0.002), pulmonary hypertension (*p* = 0.018), and hsPDA (*p* = 0.040) than non-transfused neonates or neonates receiving only CB-RBCs. In total, 25 adverse events were reported as fatal, with more events concurring to cause death: sepsis (6), bradycardia (6), acute renal failure (3), pulmonary hemorrhage (3), apnea (2), pneumonia (1), pneumothorax (1), NEC (1), coagulopathy (1), and tachyarrhythmia (1). There was no difference between arms A and B regarding the incidence of fatal adverse events.

The association between adverse event and previous transfusion was evaluated in 285 out of the 336 transfusion episodes. No adverse events were certainly due to previous transfusions, whereas 8 cases were reported as possible and five as likely connected to transfusions (eFigure 1). The patient charts were individually reviewed to determine which types of RBC products were involved. The transfusions preceding the adverse events always consisted of A-RBCs, including an IVH in a patient in the arm B (CB-RBCs) who received also A-RBCs. Taken together, these results confirm that CB-RBC transfusions in ELGANs are at least as safe as standard RBC transfusions and do not increase the incidence or severity of clinical complications arising during the clinical course of these patients.

### Transfusion needs, efficacy, and post-transfusion biochemical parameters

Forty-two of 58 patients (72.4%) required transfusions: 20 (71.4%) in the control arm and 22 (73.3%) in the experimental arm (*p* = 0.990); six of them also received erythropoietin (*p* = 0.334). A total of 153 RBC transfusions were administered, 72 in arm A and 81 in arm B, with similar transfusion needs in the two groups (3.0, IQR 1.0-5.7 and 3.0, IQR 1.0–7.0 RBC units in arm A and B, and respectively, *p* = 0.867). Transfusion rate was similar in patients receiving or not erythropoietin (1.5, IQR 1.0–3.5 and 3.5, IQR 1.0–6.0, in patients receiving or not erythropoietin, respectively, *p* = 0.163). Among 22 transfused patients in arm B, 8 received exclusively CB-RBCs, 10 both RBC types, and 4 exclusively A-RBCs (Fig. 1B and eTable 2). PMA at first transfusion, indications to be transfused, and intervals between consecutive transfusions were also similar (eTable 2). In terms of storage duration, RBC content, transfused dose, and effects on hematological and biochemical parameters, CB-RBC units were significantly older than A-RBC units (3 days, IQR 2–5 and 8 days, IQR 6–12, in A-RBCs and CB-RBCs, respectively, *p* < 0.001), had a lower Hct (61.6%, IQR 5.7–72.2 and 57.1%, IQR 53.0-62.3, in A-RBCs and CB-RBCs, respectively, *p* = 0.009), and elicited a lower hematocrit increment (Δ Hct, 12.0, IQR 9.1–16.5, and 10.4 IQR 7.5–14.0, in A-RBCs and CB-RBCs, respectively, *p* = 0.038). However, we found no correlation between ΔHct and storage duration (either with CB-RBCs or A-RBCs), so that the lower Hct increment after CB-RBCs should likely be ascribed to the lower RBC content of these units. Nonetheless, the interval between transfusions was similar after CB-RBCs and A-RBCs. Moreover, serum potassium and lactate levels, as well as pH values recorded within 24 h after CB-RBC or A-RBC transfusions, were comparable (eTable 3). In total, 230 CB units were processed into CB-RBC concentrates during the study period, but only 21.3% were transfused. eTable 4 provides characteristics of CB units at collection, after processing, and the hemolysis rate at the end of the storage.

**Table 2:** Adverse events in the 58 patients who ended the follow-up. All events that were undefinable, unlikely, possible, likely, or definitely connected with transfusions are listed. One single patient could have more than one adverse event. For all adverse events, both incidence and severity were evaluated. P_1_ indicates statistical significance of the observed differences between arms in the adverse event rate, whereas P_2_ refers to the observed differences in the adverse event severity

Adverse event type	All adverse events (*n* = 336)	Adverse events in Arm A (*n* = 165)	Adverse events in Arm B (*n* = 171)	*P*_1_	Severity assessment *P*_2_
Patients with adverse events	54 (94.9%)	27 (96.5)	27 (93.3)	0.668	-
Events per patient (median, IQR)	4 (3–8)	5 (3–7)	4 (3–8)	0.506	-
Apnea	40 (11.9)	21 (12.7)	19 (11.1)	0.739	0.608
Convulsions	8 (2.4)	3 (1.8)	5 (2.9)	0.723	1.000
IVH	15 (4.5)	8 (4.8)	7 (4.1)	0.796	0.837
PHH	2 (0.6)	1 (0.6)	1 (0.6)	1.000	NE
hsPDA	31 (9.2)	15 (9.1)	16 (9.4)	1.000	0.415
Bradycardia	34 (10.1)	18 (10.93)	16 (9.4)	0.718	0.649
Systemic hypotension	13 (3.9)	5 (3.0)	8 (4.7)	0.574	0.469
Sepsis^a^	44 (13.1)	23 (13.9)	21 (12.32)	0.746	0.403
Urinary infections	9 (2.7)	3 (1.8)	6 (3.5)	0.502	NE
Meningitis	1 (0.3)	0	1 (0.6)	1.00	ND
Pneumonia	12 (3.6)	8 (4.8)	4 (2.3)	0.250	0.367
Pulmonary hemorrhage	3 (0.9)	2 (1.2)	1 (0.6)	0.617	NE
Pulmonary hypertension	10 (3.0)	3 (1.8)	7 (4.1)	0.337	1.000
Pneumothorax	6 (1.8)	2 (1.2)	4 (2.3)	0.685	0.285
Hyaline membrane disease	35 (10.4)	17 (10.3)	18 (10.5)	1.000	0.211
NEC	5 (1.5)	1 (0.6)	4 (2.3)	0.371	NE
Acute renal failure	6 (1.8)	4 (2.4)	2 (1.2)	0.441	0.400
Surgery	14 (4.2)	3 (1.8)	11 (6.4)	0.052	NE
*Post-hemorrhagic hydrocephalus*	2 (0.6)	0	2 (1.2)	0.498	
*Patent ductus arteriosus*	2 (0.6)	0	2 (1.2)	0.498	
*Abdominal surgery*	8 (2.4)	2 (1.2)	6 (3.5)	0.283	
*Other surgery*	2 (0.6)	1 (0.6)	1 (0.6)	1.000	
Jaundice	35 (10.4)	19 (11.5)	16 (9.4)	0.593	0.971
Others^b^	13 (3.9)	9 (5.1)	4 (2.3)	0.164	0.306

Data are given as N (%) except for the number of events per patient (median (IQR). ^a^isolates were reported in 26 cases ^b^Other adverse events include thrombocytopenia (2) chylothorax (2), coagulopathy (1), supraventricular tachycardia (3), and conjunctivitis (1) in arm A end hyperglycaemia (1), thrombocytopenia (1), leukomalacia (1), and cardiomyopathy (1) in arm B. NE indicates not evaluated for the low number of events with different severity. IVH: intraventricular hemorrhage, PHH: post-hemorrhagic hydrocephalus; hsPDA: hemodynamically significant patent ductus arteriosus; NEC: necrotizing enterocolitis

### RBC transfusions and ROP

The efficacy of CB-RBC transfusions in preventing severe ROP was preliminarily assessed in 44 evaluable patients (Fig. 1B). Twenty-five patients (56.8%, 95%CI 42.2–70.3) had any stage of ROP, 10 (22.7%, 95% CI 12.8–36.9) had severe ROP, and 9 (20.4% 95%CI 11.1–34.5) required treatment. Therapy consisted of one or more administrations of anti-vascular endothelial growth factor (VEGF), combined with laser therapy (six cases) or vitrectomy (one case). All but one patient with severe ROP had received transfusions. The clinical course of the study population was typical for critically ill preterm neonates: 17 (38.6%) had IVH, 10 (22.7%) developed BPD, 4 (9.1%) surgical NEC, and 19 (43.2%) hsPDA. All but two patients experienced sepsis, 22 (50%) required inotropic agents, and all received oxygen therapy. Thirty-six patients (81.8%) needed invasive ventilation and 38 (86.3%) non-invasive ventilation. Erythropoietin was administered to 7 patients (15.9%) and 34 (77.3%) received RBC transfusions before discharge.

According to the arm allocation (intention-to-treat set), 14 (70.0%) out of 20 patients in the arm A and 19 (79.2%) out of 24 in the arm B received transfusions, with similar transfusion needs. Due to CB-RBC unavailability, ten neonates (52.6% of transfused patients) in arm B received both A-RBC and CB-RBC units and three patients (15.7%) exclusively received A-RBC units. The proportions of patients developing ROP (of any stage), severe ROP, or ROP requiring treatment in arm A and arm B were comparable (eTable 5). The two arms also did not differ for any other reported outcomes. Similar results were obtained excluding from the analysis non-transfused patients (data not shown).

Considering the high rate of protocol deviations in arm B, we further analyzed the data according to the per-protocol set. We categorized patients according to the types of RBC units received before the PMA of 32 weeks (treatment period). Thirteen neonates did not receive transfusions, 15 received only A-RBCs, 6 only CB-RBCs, and 10 both types of RBCs (Fig. 1B). Severe ROP occurred in one out of 13 non-transfused patients (7.7%), in 5 of 15 receiving exclusively A-RBCs (30.0%), and in 4 of 10 transfused with both RBC types (40.0%). Severe ROP was not observed among infants receiving only CB-RBCs (*p* = 0.107).

**Fig. 2:**
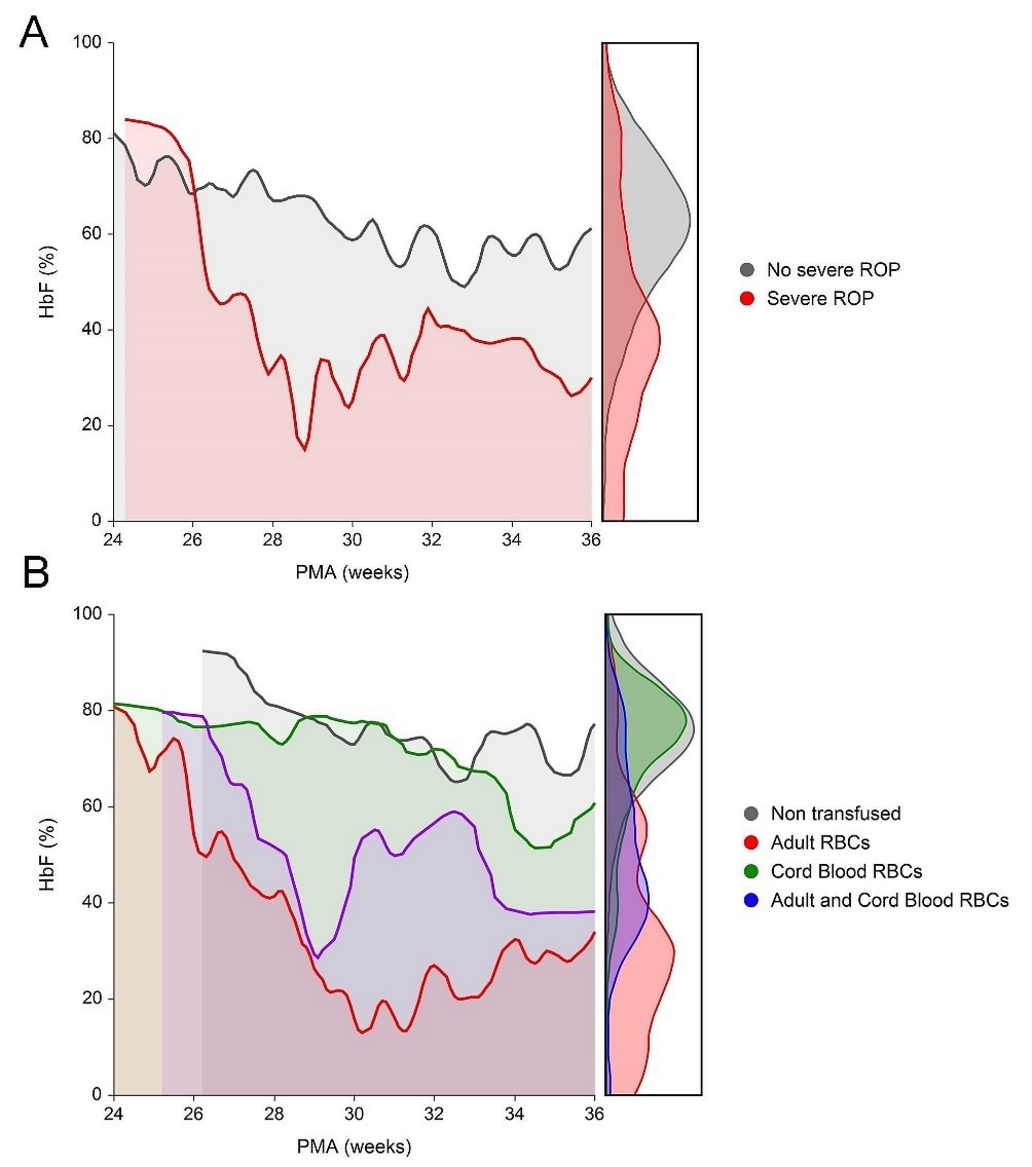
Area under the curve (AUC) of HbF and post-menstrual age (PMA). The lines represent median values of HbF. (**A**) HbF AUC of patients grouped according to the occurrence of severe ROP. (**B**) HbF AUC of patients grouped according to the types of RBC products received before 30 weeks of PMA

The AUC of HbF at various weeks of PMA was compared for patients with and without severe ROP. The median AUC was 649 (IQR 462–778) for patients without severe ROP and 498 (IQR 259–597) for those with severe ROP (*p* = 0.035) (Fig. 2A). The AUC profiles of patients who did or did not develop severe ROP, differed mainly in the earliest weeks of life. Therefore, we focused on to the type of RBC units received before the PMA of 30 weeks. In total, 16 patients were not transfused, 8 received only CB-RBCs, 15 received only A-RBCs, and 5 received both RBC types. Figure 2B illustrates the AUC of HbF in these groups. Among infants transfused exclusively with CB-RBCs, none developed severe ROP, whereas 2 non-transfused patients (12.5%), 4 patients in the A-RBC group (26.7%), and 4 (80%) receiving both A-RBCs and CB-RBCs did develop this complication (*p* = 0.005). Considering only transfused patients, 8 of 20 (40.0%) receiving A-RBCs before the PMA of 30 weeks developed severe ROP, with a relative risk of 1.66 (95% CI 1.06–2.20) as compared to those receiving only CB-RBCs (*p* = 0.040). Table 3 shows that neonates receiving only CB-RBCs have GA, birth weight, Apgar, and CRIB scores, transfusion burden, rates of IVH, sepsis, NEC, and BPD as neonates receiving A-RBCs. Apart from a slightly shorter duration of oxygen therapy, they experienced similar periods of invasive or non-invasive ventilation, and more frequently required inotropic medications (Table 3). Collectively these data suggest that transfusing ELGANs with HbF-enriched RBCs in the first weeks of life may protect them from severe ROP.

**Table 3:** Main parameters at birth and clinical characteristics of patients grouped according to the types of RBC products received within the PMA of 30 weeks

	A-RBCs *n* = 20	CB-RBCs *n* = 8	*P*
**Gestational age**,** weeks**	25.6 (25.0-26.1)	25.5 (24.6–26.8)	0.838
**Weight**,** gr**	730 (685–841)	760 (667–900)	0.575
**Male / Female**,	15 (75.0) /5 (25.0)	4 (50.0) /4 (50.0)	0.200
**Apgar score 1 min**	6 (4–7)	6 (4–7)	0.937
**Apgar score 5 min**	8 (8–9)	8 (8–9)	0.741
**CRIB II score**	12 (10–13)	11 (10–13)	0.420
**Hb at birth**,** g/dL**	14.6 (13.9–16.3)	15.0 (14.1–17.3)	0.420
**Number of transfusions**	5 (2–7)	4 (3–6)	0.455
**Erythropoietin**	2 (10.0)	0	0.353
**hsPDA**	8 (40.0)	4 (50.0)	0.629
**Sepsis**	20 (100.0)	8 (100.0)	-
**Inotropic drugs**	4 (20.0)	5 (62.5)	0.029
**IVH (all stages)**	9 (45.0)	2 (25.0)	0.327
**BPD (all stages)**	4 (20.0)	1 (12.5)	0.639
**ROP (all stages)**	14 (70.0)	5 (62.5)	> 0.999
*Stage ≥3 ROP*	8 (40.0)	0	0.040
**NEC**	2 (10.0)	2 (25.0)	0.305
**Invasive ventilation**	18 (90.0)	7 (72.5)	0.846
**Non-invasive ventilation**	16 (80.0)	6 (75.0)	0.770
**Oxygen therapy**	20 (100)	8 (100)	-
**Oxygen therapy**,** days**	82 (49–103)	32 (15–62)	0.050

CRIB II score: Clinical Risk Index for Babies II score; IVH: intraventricular hemorrhage, BPD: bronchopulmonary dysplasia; ROP: retinopathy of prematurity; NEC: necrotizing enterocolitis; Invasive ventilation included high frequency oscillatory ventilation and synchronized intermittent mandatory ventilation; Non-invasive ventilation included continuous positive airway pressure and high flow nasal cannula

To further corroborate these findings, we used logistic regression analysis to explore if A-RBC or CB-RBC transfusions exerted a diverging effect on severe ROP development. The model included severe ROP as the dependent variable and the number of A-RBC and CB-RBC transfusions before 30 week-PMA as independent variables. After adjusting for covariates with recognized effects on ROP (i.e., GA, BW, number of days on oxygen therapy), we found that the number of A-RBC, but not CB-RBC transfusions significantly predicted severe ROP, with an OR of 1.90 for each unit of A-RBCs transfused (95% CI 1.13–3.17, *p* = 0.014).

## Discussion

Repeated standard transfusions of preterm neonates produce progressive non-physiological replacement of HbF by HbA [15, 16]. Transfusing CB-RBC concentrates instead of RBC units obtained from adult donors limits HbF decrements [19]. The BORN trial is currently investigating if preserving HbF levels by transfusing CB-RBCs may limit the development of severe forms of ROP. This pre-specified interim analysis demonstrates that transfusing CB-RBCs in ELGANs is safe and can be used to treat anemia in these patients. Tissue establishments worldwide collect umbilical cord blood as a source of hematopoietic stem cells for transplanting patients with hematological diseases [29]. The use of cord blood for routine transfusion purposes has been largely reported in low-income countries, where health resources are limited and the blood supply does not meet the population’s needs [30, 31]. More recently, umbilical cord blood has attracted attention as a source of HbF-containing RBCs, to prevent preterm neonates who need transfusions from untimely exposure to HbA [32]. Nevertheless, the safety of this transfusion approach was only assessed in initial pilot studies and remained to be explored in larger populations [19]. Apart from the predominant type of hemoglobin, adult and cord blood RBCs differ in other characteristics, ranging from shape and size, to membrane composition, cell metabolism, and rheological properties. The main concerns in using allogeneic CB-RBCs to transfuse preterm neonates are related to the higher rate of hemolysis during the storage in comparison to A-RBCs, placing neonates, particularly preterm neonates, at risk for hyperkaliemia, oxidative stress, and acidosis [33, 34]. The CB-RBCs transfused in the BORN trial are collected according to criteria defined by the Italian public cord blood bank network. The CB-RBCs are filter leukodepleted and prepared using a standardized protocol designed to meet the same quality standards as A-RBC products. However, because CB-RBC units must be proven negative for fungal and bacterial contamination before distribution, the CB-RBCs transfused in this study had longer storage durations than A-RBC units. Nonetheless, the post-transfusion biochemical parameters were comparable using these two different products and the incidence and severity of adverse events were similar or even lower in patients exclusively receiving CB-RBCs. Similarly, no adverse events were definitively ascribed to transfusion and all adverse events with possible or likely connections to a previous blood product administration were seen only after A-RBC transfusions. Although this might be partly due to the higher number of A-RBC units transfused, the comprehensively collected findings allow us to conclude that CB-RBC units, as prepared and used in the BORN trial, are at least as safe as A-RBC units. Notably, the multicenter design of this study suggests that the fractionation method can be easily implemented to pursue a CB-RBC transfusion strategy. Nevertheless, the small volumes of the cord blood units that are typically collected might result in CB-RBC concentrates with lower amounts of hemoglobin as compared to A-RBCs, and this needs to be considered at transfusion. One disappointing issue emerging from this study is the difficulty of supporting the transfusion needs of patients in the experimental arm. In nearly 40% of cases, CB-RBC units were unavailable and were substituted with A-RBC units. This made comparing severe ROP rate in the two arms unfruitful. Likewise, only a minor portion of processed CB units led to CB-RBC transfusions; this suggests reorganizing CB unit procurement may be necessary to meet the ELGAN transfusion needs.

ROP is a multifactorial disease, whose pathogenesis relies on two consecutive phases affecting retinal vessel development in opposite ways [7]. The first phase occurs between 22 and 30 weeks of PMA, and is characterized by the attenuation of retinal vessels due to the hyperoxic post-natal environment. The second phase occurs between 31 and 36 weeks of PMA, and is characterized by rapid vessel growth and neovascularization stimulated by VEGF and other pro-angiogenic factors [7]. Modifying conditions that exacerbate hyperoxia in the first phase, or hypoxia in the second one, might reduce the risk for ROP development and progression [35]. Among modifiable risk factors, research has focused on the role of RBC transfusions and premature exposure to the adult form of hemoglobin, which has lower oxygen affinity than HbF. Using continuous near-infrared spectroscopy (NIRS) monitoring, we previously showed that CB-RBC and A-RBC transfusions are associated with different kinetics of cerebral regional oxygen saturation (crSO2) and cerebral fraction of tissue oxygen extraction (cFTOE) [36]. In particular, A-RBC transfusions resulted in higher crSO2 and lower cFTOE than CB-RBC transfusions, consistent with greater oxygen delivery to cerebral tissues [36]. These findings strongly support the rationale of transfusing HbF-enriched CB-RBCs, instead of RBC concentrates from adult donors to protect against ROP. The HbF trends in Fig. 2A suggests that, when patients face prolonged exposure to low HbF levels before 30 weeks of PMA, the subsequent HbF increase fails to protect them against ROP progression. Hence, the greatest impact of hyperoxia on the immature retina likely occurs at lower PMA. In our patients, A-RBC, but not CB-RBC transfusions predicted severe ROP, and severe ROP did not occur in neonates receiving only CB-RBCs before 30 week-PMA, despite similar baseline characteristics and clinical course. Taken together, these findings support reserving the limited numbers of CB-RBC units for ELGANs from birth to the age of 30 weeks, during which time the hyperoxic load could inhibit retinal angiogenesis.

The data in this interim analysis comprise only a part of the planned study sample size of 146 patients; therefore, a protective effect of CB-RBC transfusions from ROP progression could not be conclusively assessed. The study has additional limitations. First, the evidence of the highest rate of severe ROP among patients receiving both A-RBCs and CB-RBCs, is in contrast with the presumed protective role of CB-RBCs. Nevertheless, the transfusion burden of these patients was higher than in other groups (on average 7 RBC units per patient, IQR 5–7), explaining why it could not be covered exclusively by CB-RBC units. Definitely, this finding emphasizes the connection between severe ROP and transfusions, and suggests that additional risk factors may have played a role in promoting ROP progression [35]. Second, the low number of patients evaluated prevented adjusting for any center-related variability, and possible confounders due to local practices could not be excluded. In addition, the evidence of protective effects of CB-RBC transfusions on severe ROP development emerged only at “per-protocol” set of analysis, which is more prone to bias than the “intention-to-treat” set, making the results less reliable. Finally, the impact of CB transfusions on other clinical outcomes such as BPD or NEC has not been extensively investigated at this early stage of the study.

Overall, this is the first randomized trial on allogeneic CB-RBC transfusions in preterm neonates and, albeit preliminary, these data may provide clues for the design of future randomized studies. In this regard, we decided to amend the protocol of the ongoing BORN trial, by reducing the treatment period in the experimental arm until a PMA of 30 weeks, prompted by the behavior of HbF levels in patients with severe ROP. Hopefully, this change will result in improved availability of HbF-enriched-RBCs for younger neonates, resulting in fewer protocol deviations.

## Conclusions

BORN is the first randomized trial comparing CB-RBC and A-RBC transfusions to reduce severe ROP in ELGANs. The analysis of data collected in the first 58 patients proved the safety of this approach. Despite a high rate of protocol deviations, results at per-protocol analysis suggested the potential efficacy for this transfusion approach. The final analysis of the entire planned population, as well as further studies with larger sample size, are needed to confirm these promising results and support the use of new strategies to reduce ROP incidence.

### Electronic supplementary material

Below is the link to the electronic supplementary material.

Supplementary Material 1

Supplementary Material 2

## Data Availability

Data supporting the findings of this study are available in anonymized form upon reasonable request to the principal investigators.

## Declarations

### Data access, responsibility, and analysis

LT, PP, TP and IM had full access to all the data in the study and take responsibility for the integrity of the data and the accuracy of the data analysis.

## Abbreviations

ROP: Retinopathy of prematurity
HbF: Fetal hemoglobin
HbA: Adult hemoglobin
CB: Cord blood
RBC: Red blood cell
CB-RBC: Cord blood-RBC
A-RBC: Adult donor-RBC
PMA: Postmenstrual age
ELGANs: Extremely low gestational age neonates
GA: Gestational age
CRIB-II: Clinical Risk Index for Babies-II score
hsPDA: Hemodynamically-significant patent ductus arteriosus
NEC: Necrotizing enterocolitis
BPD: Bronchopulmonary dysplasia
IVH: Intraventricular hemorrhage
NICU: Neonatal intensive care unit
IQR: Interquartile range
CI: Confidence interval
OR: Odd ratio
AUC: Area under the curve
