# Supplementary material for: Cord blood transfusions in extremely low gestational age neonates to reduce severe retinopathy of prematurity: results of a prespecified interim analysis of the randomized BORN trial
Source: Ital J Pediatr. 2024 Aug 7;50:142. doi: 10.1186/s13052-024-01714-w (PMC11305044; doi:10.1186/s13052-024-01714-w)
Supplement: Supplementary file 1 — Supplementary Material 1 [file 13052_2024_1714_MOESM1_ESM.doc]

**eFigure 1**. Adverse events possibly (empty symbols) or likely (solid symbols) imputable to transfusions. The intraventricular hemorrhage observed in a patient allocated to arm B occurred after an A-RBC unit transfusion. The protocol deviation was due to the unavailability of ABO/RhD-matched CB-RBC units.

**
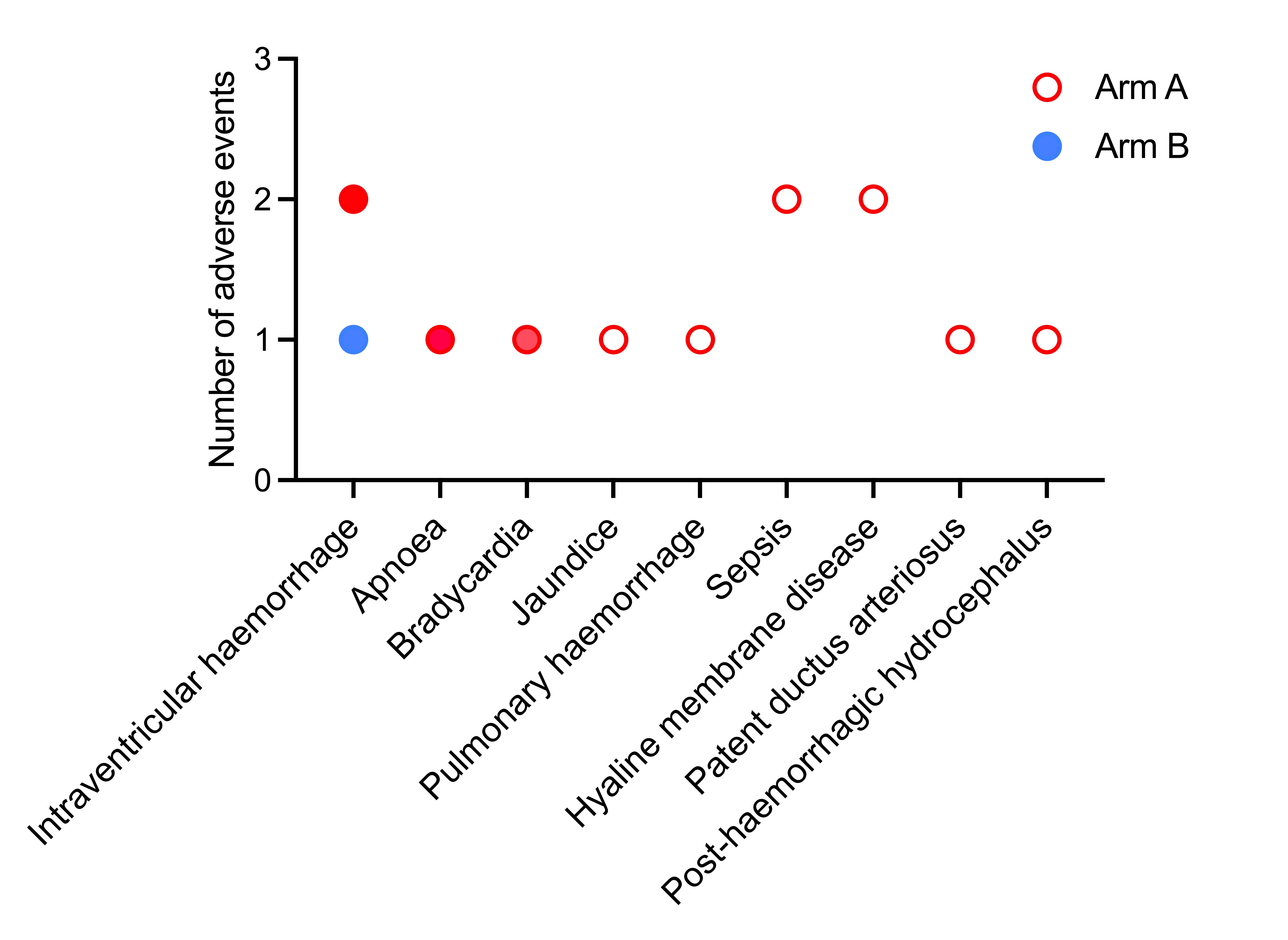
**
