# Supplementary material for: Cord blood transfusions in extremely low gestational age neonates to reduce severe retinopathy of prematurity: results of a prespecified interim analysis of the randomized BORN trial
Source: Ital J Pediatr. 2024 Aug 7;50:142. doi: 10.1186/s13052-024-01714-w (PMC11305044; doi:10.1186/s13052-024-01714-w)
Supplement: Supplementary file 2 — Supplementary Material 2 [file 13052_2024_1714_MOESM2_ESM.doc]

| **eTable 1.** Adverse events recorded in 58 patients censored according to transfusions received before the age of 32 post- menstrual weeks**.** | | | | | | | | | |
| --- | --- | --- | --- | --- | --- | --- | --- | --- | --- |
|  | **Non-Tranfused**  **N = 18** | **%** | **A-RBC**  **N = 22** | **%** | **CB-RBC**  **N = 8** | **%** | **A-RBC/ CB-RBC**  **N = 10** | **%** | **p value** |
| **Total adverse events** | 62 |  | 161 |  | 30 |  | 84 |  | NA |
| **Patients with AE** | 16 | 88.8 | 22 | 100 | 8 | 100 | 8 | 80 | 0.373 |
| **AE per patient (Median IQR)** | 4 (2-4.2) |  | 6(4-10) |  | 4 (3-4) |  | 7.5 (2.2-12.2) |  | **0,014** |
| **Apnoea** | 5 | 8,1 | 20 | 12,4 | 6 | 20,0 | 9 | 10,7 | 0,406 |
| **Convulsions** | 0 | 0,0 | 4 | 2,5 | 0 | 0,0 | 4 | 4,8 | 0,229 |
| **IVH** | 2 | 3,2 | 7 | 4,3 | 1 | 3,3 | 5 | 6,0 | 0,859 |
| **PHH** | 1 | 1,6 | 1 | 0,6 | 0 | 0,0 | 1 | 1,2 | 0,836 |
| **hsPDA** | 8 | 12,9 | 13 | 8,1 | 4 | 13,3 | 6 | 7,1 | 0,512 |
| **Bradycardia** | 3 | 4,8 | 18 | 11,2 | 4 | 13,3 | 9 | 10,7 | 0,479 |
| **Systemic hypotension** | 2 | 3,2 | 6 | 3,7 | 1 | 3,3 | 4 | 4,8 | 0,947 |
| **Sepsis** | 7 | 11,3 | 23 | 14,3 | 4 | 13,3 | 10 | 11,9 | 0,972 |
| **Urinary infections** | 2 | 3,2 | 1 | 0,6 | 1 | 3,3 | 5 | 6,0 | 0,102 |
| **Meningitis** | 0 | 0,0 | 0 | 0,0 | 0 | 0,0 | 1 | 1,2 | 0,388 |
| **Pneumonia** | 0 | 0,0 | 8 | 5,0 | 0 | 0,0 | 4 | 4,8 | 0,196 |
| **Pulmonary haemorrhage** | 1 | 1,6 | 1 | 0,6 | 1 | 3,3 | 0 | 0,0 | 0,350 |
| **Pulmonary hypertension** | 3 | 4,8 | 4 | 2,5 | 1 | 3,3 | 2 | 2,4 | 0,801 |
| **Pneumothorax** | 2 | 3,2 | 2 | 1,2 | 1 | 3,3 | 1 | 1,2 | 0,662 |
| **Hyaline membrane disease** | 10 | 16,1 | 17 | 10,6 | 3 | 10,0 | 5 | 6,0 | 0,263 |
| **NEC** | 1 | 1,6 | 2 | 1,2 | 0 | 0,0 | 2 | 2,4 | 0,804 |
| **Acute renal failure** | 0 | 0,0 | 5 | 3,1 | 0 | 0,0 | 1 | 1,2 | 0,327 |
| **Surgery** | 1 | 1,6 | 4 | 2,5 | 1 | 3,3 | 8 | 9,5 | **0,040** |
| ***Post-haemorrhagic hydrocephalus*** | 0 | 0,0 | 0 | 0,0 | 1 | 3,3 | 1 | 1,2 | 0,433 |
| ***Patent ductus arteriosus*** | 1 | 1,6 | 0 | 0,0 | 0 | 0,0 | 1 | 1,2 | 0,433 |
| ***Abdominal surgery*** | 0 | 0,0 | 3 | 1,9 | 0 | 0,0 | 5 | 6,0 | 0,070 |
| ***Other surgery*** | 0 | 0,0 | 1 | 0,6 | 0 | 0,0 | 1 | 1,2 | 0.433 |
| **Jaundice** | 11 | 17,7 | 17 | 10,6 | 2 | 6,7 | 5 | 6,0 | 0,120 |
| **Others** | 3 | 4,8 | 8 | 5,0 | 0 | 0,0 | 2 | 2,4 | 0,494 |
| **Deaths** | 5 | 27.7 | 7 | 31.8 | 2 | 25 | 0 | 0 | 0.371 |

**eTable 2.** Transfusion-related characteristics of the 58 patients included in the interim analysis.

|  | **Arm A**  **n = 28** | **Arm B**  **n = 30** | ***P*** |
| --- | --- | --- | --- |
| Transfused patients | 20 (71.4) | 22 (73.3) | >0.990 |
| RBC units per patient | 3.0 (1.0-5.7) | 3.0 (1.0-7.0) | 0.867 |
| *1 unit* | 6 (30.0) | 7 (31.8) | 0.593 |
| *2 units* | 4 (20.0) | 2 (9.1) |
| *>3 units* | 10 (50.0) | 13 (59.1) |
| Patients receiving only A-RBCs | 20 (100) | 4 (18.2) | <0.001 |
| Patients receiving both A-RBC and CB-RBCs | 0 | 10 (33.3) |
| Patients receiving only CB-RBCs | 0 | 8 (26.6) |
| PMA at first transfusion, weeks | 27.5 (25.8-29.6) | 27.8 (26.1-29.4) | 0.860 |
| Transfusion-free survival, days | 9.5 (4.2-25.2) | 13.5 (6.0-23.5) | 0.705 |
| Interval between transfusions, days | 6.0 (3.0-10.0) | 7.0 (3.0-11.7) | 0.828 |
| RhD negative patients | 2 (10.0) | 3 (13.6) | 0.716 |
| Total transfusions, any type | 72 | 81 | <0.001 |
| Total A-RBC transfusions | 72 (100.0) | 32 (39.5) |
| Total CB-RBCs | 0 | 49 (60.5) |
| Indications to transfuse |  |  |  |
| *Acute anemia* | 46 (64.8) | 50 (64.9) | 0.994 |
| *Chronic anemia* | 22 (31.0) | 24 (31.2) |
| *Surgery* | 3 (4.2) | 3 (3.9) |

Data are expressed as N (%) or Median (IQR).

**eTable 3.** Storage, transfusion efficacy, and effects on biochemical parameters of A-RBC and CB-RBC transfusions.

|  | **A-RBC**  **(n = 104)** | **CB-RBC**  **(n = 49)** | **P value** | **Missing (%)** |
| --- | --- | --- | --- | --- |
| Storage duration, days | 3 (2-5) | 8 (6-12) | <0.001 | 16 (10.4) |
| Transfusion dose, mL/Kg | 19.2 (15.3-20.1) | 18.2 (15.0-20.2) | 0.945 | 5 (3.3) |
| RBC unit Hct, % | 61.6 (57.7-72.2) | 57.1 (53.0-62.3) | 0.009 | 97 (63.4) |
| Pre-transfusion Hct, % | 28.6 (25.4-32.0) | 28.0 (25.1-31.0) | 0.690 | 7 (4.5) |
| Post-transfusion Hct, % | 41.0 (37.1-46.0) | 38.0 (34.1-43.8) | 0.027 | 11 (7.2) |
| Δ Hct, % | 12.0 (9.1-16.5) | 10.4 (7.5-14.0) | 0.038 | 12 (7.8) |
| Between-transfusion interval, days | 6.0 (3.0-10.5) | 4.0 (2.0-10.5) | 0.199 | 0 |
| Post-transfusion, pH | 7.32 (7.27-7.37) | 7.30 (7.26-7.35) | 0.198 | 10 (6.5) |
| Post-transfusion lactate, mmol/L | 1.1 (0.8-1.7) | 0.9 (0.6-1.4) | 0.138 | 17 (11.1) |
| Post-transfusion potassium, mEq/L | 4.3 (3.9-4.7) | 4.1 (3.7-4.6) | 0.570 | 12 (7.8) |

**eTable 4.** Characteristics of 230 CB units fractionated into RBC concentrates during the study period.

| Produtc | Timing | Parameter | Median | 25 percentile | 75 percentile |
| --- | --- | --- | --- | --- | --- |
| Cord blood | At collection | Volume (mL) | 86,00 | 76,00 | 98,00 |
|  |  | Hematocrit | 36,40 | 34,70 | 38,93 |
|  |  | RBC mass (ml) | 32 | 27 | 37 |
|  |  | WBC count (x10^3^/μL) | 10,20 | 8,79 | 11,84 |
|  |  | Platelet count (x10^3^/μL) | 218 | 190 | 254 |
| RBC concentrates | After fractionation* | Volume (ml) | 38,00 | 30,00 | 46,50 |
|  |  | Hematocrit | 56,40 | 53,00 | 59,60 |
|  |  | RBC mass (ml) | 21,00 | 16,00 | 27,00 |
|  |  | RBC mass recovery (%) | 65,6 | 59,1 | 74,1 |
|  |  | WBC (x10^6^) content | 0,00 | 0,00 | 0,00 |
|  |  | Platelets count (x10^3^/μL) | 1,45 | 0,83 | 2,62 |
|  |  | Platelets reduction (%) | 80,00 | 70,00 | 90,00 |
| RBC concentrates | Day 15** | Hemolysis rate (%) | 0,16 | 0,00 | 0,23 |

* Units are sampled after adding 1:3 volume of SAG-M. ** Since older units cannot be irradiated, CB-RBCs are stored for no more than 2 weeks. Hemolysis rate was evaluated as the percentage of free hemoglobin on the initial total hemoglobin content and was evaluated in those units not selected for transfusion.

**eTable 5**. Main parameters at birth and clinical characteristics of 44 patients evaluated for ROP.

|  | **Arm A n = 20** | **Arm B n = 24** | ***P*** |
| --- | --- | --- | --- |
| **Gestational age, weeks** | 26.1 (25.4-27.2) | 26.43 (25.0-27.4) | 0.962 |
| **Weight, gr** | 765 (702-956) | 872 (705-957) | 0.516 |
| **Male / Female** | 11 (55.0) /9 (45.0) | 14 (58.3) /10 (41.7) | 0.824 |
| **Transfused patients** | 14 (70.0) | 19 (79.2) | 0.484 |
| *Only A-RBC* | 14 (100) | 3 (15.8) | <0.001 |
| *Only CB-RBC* | 0 | 6 (31.6) |
| *A-RBC and CB-RBC* | 0 | 10 (52.6) |
| **Number of transfusions** | 4 (2-7) | 3 (2-7) | 0.853 |
| **Sepsis** | 20 (100) | 22 (91.7) | 0.492 |
| **IVH (any stage)** | 7 (35.0) | 10 (41.7) | 0.760 |
| *Stage 3 IVH* | 3 (15.0) | 3 (12.5) | 0.953 |
| *Stage 4 IVH* | 1 (5.0) | 1 (4.1) |
| **BPD (any stage)** | 5 (25.0) | 5 (20.8) | 0.742 |
| *Stage 3 BPD* | 2 (10.0) | 1 (4.2) | 0.583 |
| **ROP (any stage)** | 12 (60.0) | 13 (54.2) | 0.766 |
| *stage I ROP* | 1 (5.0) | 1 (4,2) | 0.898 |
| *stage 2 ROP* | 7 (35.0) | 6 (25.0) |
| *Stage ≥ 3 ROP* | 4 (20.0) | 6 (25.0) |
| **ROP treatment** | 4 (20.0) | 5 (20.8) | 0.894 |
| **NEC** | 1 (5.0) | 3 (12.5) | 0.388 |
| **Invasive ventilation** | 18 (90.0) | 18 (75.0) | 0.111 |
| **Non-invasive ventilation** | 18 (90.0) | 20 (70.8) | 0.198 |
| **Oxygen therapy** | 20 (100) | 24 (100) | - |
| **Oxygen therapy, days** | 73 (20-97) | 47 (18-78) | 0.345 |

IVH: intraventricular hemorrhage, BPD: bronchopulmonary dysplasia; ROP: retinopathy of prematurity; NEC: necrotizing enterocolitis; Invasive ventilation included high frequency oscillatory ventilation and synchronized intermittent mandatory ventilation; Non-invasive ventilation included continuous positive airway pressure and high flow nasal cannula.
